# Prevalence, antimicrobial susceptibility pattern and associated risk factors for urinary tract infections in pregnant women attending ANC in some integrated health centers in the Buea Health District

**DOI:** 10.1186/s12884-021-04142-4

**Published:** 2021-10-04

**Authors:** Innocentia Nji Ngong, Jerome Fru-Cho, Melduine Akom Yung, Jane-Francis Kihla Tatah Akoachere

**Affiliations:** Department of Microbiology and Parasitology, Faculty of Science, University of Buea, South West Region Buea, Cameroon

**Keywords:** Urinary tract infection, Pregnancy, Risks factors, Antibiotic susceptibility

## Abstract

**Background:**

Urinary tract infections (UTIs) are the second most frequent infections after respiratory tract infections that affect humans, with over 150 million cases per year. The anatomy of the female urinary tract predisposes them to UTIs than men. More so, physiological and hormonal changes during pregnancy put pregnant woman at risk of UTIs. Untreated UTI(s) in pregnancy can be detrimental to both the mother and child causing preterm labour, low birth weight and pyelonephritis. The situation is worrisome because the infection can be asymptomatic. This study investigated the prevalence and risk factors for UTIs, diagnostic potential of dipstick analyses and antimicrobial susceptibility of uropathogens from pregnant women attending ANC in some Integrated Health Centers (IHCs) in Buea Health District (BHD).

**Methods:**

A structured questionnaire was administered to consenting pregnant women at participating IHCs to collect data on demographic characteristics, risk factors and symptoms of UTI. Urine samples were collected for dipstick analysis and culture. Antibiograms were performed on the isolates by the disc diffusion method. A bivariate analysis was performed to investigate the association of the risk factors to UTI. Chi square (χ^2^) test, odds ratios with corresponding 95% confidence intervals were used to compare statistics and test for associations at a significant level of *p* ≤ 0.05.

**Results:**

Of the 287 participants recruited, 89(31%) were positive for UTI. There were 150 women with no symptoms of which 43(28.7%) were positive for UTI. *E. coli* was the most frequent (43.2%) of the organisms implicated in bacteriuria. There was no significant association between the risk factors studied and UTI. Isolates were most sensitive to ciprofloxacin (73.5%) and gentamycin (73.5%) and resistant to ceftriaxone (70.6%) and tetracycline (64.7%). Nitrite test was highly specific (100%) for the diagnosis of UTI while leucocyte esterase was more sensitive (48.3%) than specific (44.9%).

**Conclusions:**

The prevalence of UTI in BHD was high. In conformity with previous findings in same area, there were no risk factors associated with UTI. We recommended a longitudinal study with a larger sample size to follow up the women to term in order to determine the gravity of this infection on pregnancy outcomes.

## Introduction

Urinary tract infection (UTI) is the inflammation of any part of the urinary system which consists of the kidney, bladder, ureter and urethra mediated by microorganisms. In USA, about 11 million cases of UTIs are recorded yearly [1]. In Africa, the general prevalence is not well established, however, a prevalence of 65.9% in Buea and 54% in Bamenda in the South West and North West of Cameroon respectively has been recorded [2]. More women than men suffer from this infection because the anatomy of their urinary tract, which is shorter and closer to the anus and vagina, facilitating infection [3]. UTIs are the most common bacterial infections in pregnancy. The risk increases in pregnancy because the uterus which is located between the bladder and the rectum grows and narrows the bladder anteriorly thereby narrowing it thus increasing frequency of urination and limiting complete drainage of urine [4].

Hormonal changes increase urinary stasis and vesicoureteral reflux and thus favour bacterial growth [5].

Urinary tract infections in pregnant women are associated with maternal as well as foetal morbidity such as preterm labour, hypertension/preeclampsia, pyelonephritis, low birth weight and prematurity. Asymptomatic infections go unnoticed and the infection may cause cystitis and pyelonephritis [6]. In a retrospective study carried out by Njim *et al*. [7] at the Buea Regional Hospital, an incidence of 19.5% of low birth weight was recorded with infections as the leading the cause, UTIs inclusive. This was associated with foetal distress syndrome and sometimes death. In another study [8], UTI was incriminated as a significant risk factor for preeclampsia with 17% prevalence amongst women with preeclampsia. Thus UTIs are a great cause for concern with regards to reproductive and child health.

It has been reported that modern ante-natal screening combined with appropriate antibiotic treatment could significantly reduce pregnancy complications caused by urinary tract infection thus improving maternal and neonatal health [9]. It is well established that urine culture is the gold standard for diagnosing UTIs. However, culture is expensive and time consuming to be introduced as part of the antenatal consultation (ANC) routine test in resource poor countries like Cameroon. In addition, most health care facilities in such settings lack culture facilities and urine testing is usually by the dipstick test. Thus identification of the risks factors for UTI in pregnancy will reduce the prevalence, cost of diagnosis, and treatment of UTI.

A similar study in the Buea Health District conducted over 5 years ago reported a UTI prevalence of 23.5% but found no association between suspected risk factors and prevalence of UTI in pregnancy [10]. However, the sample size of that study was small (102) and not representative of the entire Buea Health District. More so the risk factors assessed were not exhaustive. For these reasons, the present study was conducted to verify if with a greater sample size, risk factors associated with urinary tract infection in pregnancy could be identified given the high prevalence.

## Materials and methods

### Study design

This was a cross sectional study in which pregnant women registered for ANC within the months of August to November 2017 in the major Integrated Health Centers of the Buea Health District and gave signed informed consent were recruited in this study. Data on the symptoms of UTIs reported, demographic characteristics, medical history and information on the risk factors tested were obtained using a questionnaire. Urine samples were collected for urine dipstick analysis and culture for the identification of bacteria. The validity of dipstick test in the screening of UTIs was calculated and antimicrobial susceptibility testing was carried out on the isolates by the disc diffusion method.

### Study area

This study was carried out in the Buea Health District, located in Buea, the capital city of South West Region of Cameroon. It is situated on latitude 4^o^.1538′ North, longitude 9^o^.2337′ East and 959 m above sea level. Buea Health District is one of the four health districts in Fako Division. With regards to its health structure, the Buea Health District is made up of 7 health areas. It has a total population of 153,827 as of 2015 [11]. The Integrated Health Centers (IHC) sampled included; Mile 16 IHC, Muea Divisional Hospital, Buea road IHC, Bova IHC, Tole IHC and Molyko IHC.

### Sample size estimation and study population

The sample size was calculated as previously described for cross sectional studies [12]. Following this method, a minimum sample size of 284 participants was obtained assuming a prevalence of 23.5% bacteriuria reported in the same area [10].

All pregnant women attending ANC in the major Integrated Health Centers in all Health Areas of the Buea Health District were included in this study. Each participant signed an informed consent form. Assent was sought from participants <21 years old and consent from their parents/guardian. Pregnant women who were on antibiotics or had taken any within the previous 2 weeks, as well as those who refused to give consent, were excluded from the study.

### Sample collection

Sterile urine samples were collected from each participant for urinalysis and urine culture. The women were given instructions on how to collect a clean catch mid-stream sterile urine sample. They were asked to clean the labia and area around the urethra with sterile water and urine collected with the labia held apart. They were to pass out urine into the toilet pot for a few seconds, then collect a portion of the mid-stream urine before emptying their bladder into the toilet pot. About 3 mL of the specimen was poured into another wide-mouth container and used for urine dipstick analysis and microscopy. The rest of the sample was kept in a cold box and transported to the Faculty of Science Clinical Diagnostic Laboratory, University of Buea within 3 h of sample collection for culture. Capillary blood from a finger prick was also collected for the determination of haemoglobin concentration using a haemoglobinometer (Mission HB haemoglobinometer, Italy).

### Sample analysis

#### Macroscopy and dipstick analysis

The color and consistency of freshly collected urine samples were recorded. A Combii 11 dipstick (URIT Medical Electronic Co-LTD) was used for urinalysis. The strip contained the following analytes; ascorbic acid, specific gravity, pH, ketone, glucose, urobilinogen, bilirubin, nitrite, leucocytes and proteins. The reagent strip pad was completely immersed in fresh urine and observed for color changes. These were read against the color chart on the labeled test strip container and the results recorded.

The sensitivity and specificity of nitrite and leucocyte esterase of the urine dipstick analyses were calculated using formulae previously described [13].

$$Sensitivity=\frac{true\ positive}{True\ positive+ false\ negative}$$

$$=\frac{\mathrm{participants}\ \mathrm{who}\ \mathrm{were}\ \mathrm{positive}\ \mathrm{by}\ \mathrm{both}\ \mathrm{culture}\ \mathrm{and}\ \mathrm{dipstick}}{\mathrm{participants}\ \mathrm{positive}\ \mathrm{by}\ \mathrm{both}\ \mathrm{culture}\ \mathrm{and}\ \mathrm{dipstick}+\mathrm{positive}\ \mathrm{by}\ \mathrm{culture}\ \mathrm{but}\ \mathrm{negative}\ \mathrm{by}\ \mathrm{dipstick}}$$

$$Specificity\frac{true\ negative}{True\ negative+ false\ positive}$$

$$=\frac{\mathrm{participants}\ \mathrm{who}\ \mathrm{were}\ \mathrm{negative}\ \mathrm{by}\ \mathrm{both}\ \mathrm{culture}\ \mathrm{and}\ \mathrm{dipstick}}{\mathrm{participants}\ \mathrm{negative}\ \mathrm{by}\ \mathrm{both}\ \mathrm{culture}\ \mathrm{and}\ \mathrm{dipstick}+\mathrm{negative}\ \mathrm{by}\ \mathrm{culture}\ \mathrm{but}\ \mathrm{positive}\ \mathrm{by}\ \mathrm{dipstick}}$$

Considering the two parameters simultaneously, the combined sensitivity and specificity were calculated thus;

$$\mathbf{Net}\ \left(\mathbf{combined}\right)\ \mathbf{sensitivity}=\left(\mathrm{sensitivity}\ \mathrm{of}\ \mathrm{leucocytes}\right)+\left(\mathrm{sensitivity}\ \mathrm{of}\ \mathrm{nitrite}\mathrm{s}\right)-\left(\mathrm{sensitivity}\ \mathrm{of}\ \mathrm{leucocytes}\ \mathrm{x}\ \mathrm{s}\mathrm{ensitivity}\ \mathrm{of}\ \mathrm{nitrite}\right)$$

$$\mathbf{Net}\ \left(\mathbf{combined}\right)\ \mathbf{specificity}=\mathrm{specificity}\ \mathrm{of}\ \mathrm{leucocytes}\ \mathrm{x}\ \mathrm{specificity}\ \mathrm{of}\ \mathrm{nitrites}.$$

### Urine culture and biochemical testing

Cysteine Lactose Electrolyte Deficient (CLED) agar (Liofilchemsrl. Roseto d. Abruzzi TE-Italy), was prepared according to manufacturer’s instructions and stored in the refrigerator at 2-8 °C. Prior to inoculation, the pre-casted culture media were removed and placed on a clean bench to warm to room temperature. Ten microliters (10 μL) of each sample was streaked on the media and incubated at 37 °C for 18–24 h, after which the number of colonies on each plate was recorded. Only samples with bacterial count ≥10^5^ colony forming unit (CFU) per mL of urine were considered positive. Colony morphology on CLED was recorded. Pure cultures were prepared by sub-culturing on nutrient agar. Smears of isolates were prepared and Gram stained [14]. The Coagulase and Catalase tests were performed on gram positive isolates while for the gram negatives, glucose and lactose breakdown were tested on KIA medium as well as indole test and API20E used for species identification.

### Antibiotic susceptibility testing

Antimicrobial susceptibility of pure isolates was tested by the disc diffusion method on Mueller-Hinton agar. The antibiotics (Cypress Diagnostics, Italy) used included: gentamycin (20 μg), ciprofloxacin (5 μg), trimethoprim (1.25 μg) and sulphamethoxazole (23.75 μg), kanamycin (30 μg), chloramphenicol (5 μg), tetracycline (30 μg), ceftriaxone (30 μg), cefatoxime (30 μg) and ampicillin (10 μg). The plates with the discs were incubated in an inverted position at 37 °C overnight. Diameters of zones of inhibition were measured using a ruler to the nearest mm and compared with recommended CLSI M100-S12 Standards [15] and the organisms reported as susceptible, intermediate, or resistant to the antimicrobial agents.

### Data analysis

Data was entered into a prepared template on Microsoft Excel 2010 and later exported to the Statistical Package for Social Sciences (SPSS) for Windows Version 18.0 (Chicago, IL, USA) and EPI Info 7.0 (CDC, USA) for analyses. Relationships between categorical variables were assessed using either the Chi Square test or the Fischer Exact test. A bivariate analysis was performed to investigate the level of association of the risk factors (exposure) to the outcome variable (Culture positivity). Ninety-five percent (95%) confidence intervals and Odds ratios (OR) were reported for key parameters under investigation to quantitate associations. OR > 1with the null value-1 lying outside the 95% confidence interval were considered as positive associations whereas OR < 1 were consider as negative association. Statistical significance was set at *p* value ≤0.05.

### Ethical considerations

Ethical clearance was granted by the Faculty of Health Sciences Institutional Review Board (FHS-IRB), University of Buea. Administrative clearances were obtained from the South West Regional Delegation of Public Health and the Buea District Health Service. Permission was obtained from the management of the participating health facilities. Every participant indicated their willingness to participate in the study by signing an informed consent form. Participants <21 years gave assent and their parents/guardians gave informed consent. Participants were free to withdraw at any stage of the research without any sanctions. All experiments were performed in accordance with the relevant guidelines and regulations. Laboratory results of urine analysis and culture were sent to the respective health centers in sealed envelopes for delivery to the participants by the Chief of Centers. Personal information of all participants was kept confidential and electronic files were pass-word protected and accessible only to authorised research group members.

## Results

Overall, 287 pregnant women of ages between 16 and 47 years (with mean age of 26.6 ± 5.3 years,) were recruited. The majority of women were within the age group 21–25 years (33.5%), had attained secondary level of education (55.8%), were married (68.3%), in the third trimester of pregnancy (56.1%), and had started ANC in the second trimester of pregnancy (Table 1).

**Table 1:** Characteristics of study participants

Parameter		n (%)
**Age**	≤ 20	34 (11.9)
	21–25	96 (33.5)
	26–30	93 (32.4)
	31–40	60 (21.0)
	>40	4 (1.4)
**Educational Level**	Illiterate	4 (1.4)
	Primary	29 (10.8)
	Secondary	159 (55.8)
	Tertiary	93 (32.6)
**Marital status**	Single	90 (31.4)
	Married	196 (68.3)
	Separated/Divorce	1 (0.4)
**Gestational age**	1st trimester	17 (6.0)
	2nd trimester	109 (38.0)
	3rd trimester	161 (56.1)
**Gravidity**	Primigravida	110 (38.3)
	Multigravida	177 (61.7)
**Parity**	Nullipara	120 (41.8)
	Multipara	167 (58.8)
Gestational age at first ANC	1st trimester	107 (37.3)
	2nd trimester	173 (60.3)
	3rd trimester	7 (2.4)
**Maternal age at first pregnancy**	≤19	52 (18.1)
	20–29	190 (66.2)
	30–39	42 (14.6)
	>40	3 (1.1)
**Sexual activity while pregnant**	Yes	231 (80.5)
	No	56 (19.5)

Significant bacteriuria was reported in 89 of the 287 participants giving a prevalence of 31.0%. One hundred and fifty (150) participants did not report any symptom of UTIs. Out of these, 43 (28.7%**)** were positive for bacteriuria (Table 2). Nine bacterial species were isolated, with *E*. *coli* as the leading cause of UTIs and accounting for 43.2% of all gram negative bacterial infections (Table 3).

**Table 2:** Prevalence of asymptomatic bacteriuria

Culture	Characteristics			
	Symptomatic	Asymptomatic	Total n (%)	χ2 (***p***-value)
**Positive** **n (%)**	46 (33.6)	43 (28.7)	89 (31.0)	0.59 (0.44)
**Negative** **n (%)**	91 (66.4)	107 (71.3)	198 (69.0)	
Total	137	150	287 (100)

**Table 3:** Prevalence of uropathogens in samples

Type of bacteria		n (%)
Gram positive	Coagulase negative Staphylococcu*s* (CONS)	5 (5.6)
	*Staphylococcus aureus*	8 (9.0)
**Total**		**13 (14.6)**
Gram	*Escherichia coli*	38 (43.2)
Negatives	*Enterobacter amnigenis*	3 (3.4)
	*Klebsiella pneumoniae*	17 (19.1)
	*Proteus mirabilis*	5 (5.6)
	*Proteus vulgaris*	7 (7.9)
	*Pseudomonas aeruginosa*	5 (5.6)
	*Klebsiella oxytoca*	1 (1.1)
Total		76 (85.4)
	Grand Total	89 (31.0)

Risks factors for UTI associated to maternal obstetric characteristics are shown on Table 4. There was no statistically significant association between these factors and UTI. However, multigravidas (OR = 1.01, 95%CI =0.60–1.68) and multiparous women (OR = 1.08, 95% CI = (0.65–1.8), advanced age (> 30) (OR = 1.2, 95%CI = 0.66–2.08) and having sexual intercourse ≤4 times in a month (OR = 1.7, 95%CI = (0.94–3.10) had high prevalence of UTI (Table 4).

**Table 4:** Demographic, obstetric and behavioral characteristics as risk factors for UTI

		Culture Status			χ2 (***p***-value)
Risk factors	Parameter	Positive n (%)	Negative n (%)	OR (95% CI)	
**Gravidity**	Primigravida	34 (38.2)	76 (38.4)	1.01 (0.60–1.68)	0.01 (0.92)
	Multigravida	**55 (61.8**)	122 (61.6)		
**Parity**	Nulliparous	36 (40.4)	54 (27.3)	1.08 (0.65–1.8)	0.03 (0.85)
	Multiparous	**53 (59.6)**	144 (72.7)		
**Gestational age**	1st trimester	8 (9.0)	9 (4.5)		2.96 (0.23)
	2nd trimester	36 (40.4)	73 (36.9)		
	3rd trimester	45 (50.6)	116 (58.6)		
**Maternal age at first pregnancy**	≤Teen age 19	**72 (80.9)**	163 (82.3)	0.9 (0.4–1.7)	0.02 (0.91)
	>Teen age 19	17 (19.1)	35 (17.7)		
**Maternal age**	< 30	63 (70.8)	147 (74.2)	1.2 (0.66–2.08)	0.37 (0.54)
	>30	**26 (29.2)**	51 (25.8)		
**Marital status**	Single/divorced	24 (27.0)	66 (33.3)	0.73 (0.42–1.28)	1.67 (0.43)
	Married	65 (73.0)	132 (66.7)		
**Level of education**	Illiterates and basic	6 (18.2)	27 (81.8)	0.46 (0.18–1.15)	3.76 (0.21)
	Above basic	83 (32.7)	171 (67.3)		
**Gestational age at first ANC**	<3 months	37 **(41.6)**	70 (35.4)	0.77 (0.46–1.28)	1.02 (0.31)
	>3 months	52 (58.4)	128 (64.4)		
**Sexual activity during pregnancy**	Yes	69 (77.5)	162 (81.8)	0.77 (0.41–1.42)	0.45 (0.49)
	No	20 (22.5)	36 (18.2)		
**Use of contraceptive**	No	74 (83.1)	169 (85.8)	0.84 (0.41–1.92)	0.79 (0.68)
	Yes	15 (16.9)	28 (14.2)		
**History of past infection**	Present	23 (30.7)	30 (19.2)	0.5 (0.62–1.68)	4.23 (0.12)
	Absent	52 (69.3)	126 (80.8)		
**Number of intercourses/month**	≤4 times	**44 (62.9)**	121 (74.2)	1.7 (0.94–3.10)	3.07 (0.07)
	>4 times	26 (37.1)	42 (25.8)		

With regards to hygiene and sanitation practices of participants, neither the type of toilet used, presence of regular water supply for those who used water cistern toilets nor the type of pants used showed statistically significant associations with the prevalence of UTI (Table 5).

**Table 5:** Hygiene and sanitation of participants as risk factors for UTI

Parameter		Positive culture	Negative culture	OR (95%CI)	χ^**2**^ (***p***-value)
**Type of toilet use**	Pit	34 (33.3)	68 (66.7)		1.29 (0.55)
	Water cistern	48 (31.2)	109 (68.3)		
	Bucket	7 (22.6)	24 (77.4)		
**Is the toilet personal or shared**	Shared	33 (28.2)	84 (71.8)	0.8 (0.48–1.33)	0.73 (0.39)
	Personal	56 (32.9)	144 (67.1)		
**Shared with how many people**	1–3	21 (37.5)	51 (42.8)		11.1 (0.68)
	4–6	23 (41.0)	35 (29.5)		
	>6	12 (21.1)	33 (27.6)		
**Convenient water supply for water cistern users**	Yes	38 (29.9)	89 (70.1)	1.025	0.003 (0.859)
	No	17 (37.8)	28 (62.2)		
**Cleaning one’s self after using the toilet**	Front to back	76 (32.6)	157 (67.4)	1.53 (0.77–3.02)	1.50 (0.22)
	Back to front	13 (24.1)	41 (76.0)		
**Type of pants used**	Nylon	11 (39.3)	17 (60.7)	1.49 (0.67–3.34)	0.59 (0.44)
	Cotton	78 (30.2)	180 (69.8)		
**Number of pants per day**	≤2	11 (39.3)	17 (60.7)	0.796 (0.87–2.42)	0.362 (0.332)
	>2	7 8 (15.3)	180 (15.3)		
**Douching after intercourse**	Yes	62 (30.4)	142 (69.2)	1.04 (0.57–1.91)	0.02 (0.88)
	No	20 (29.2)	48 (70.6)		

The association between physiological characteristics of the pregnant women and UTIs are shown on Table 6. There was a trend towards positive association between anaemia, glucosuria and the occurrence of UTI (OR = 1.08, 95%CI = 0.51–2.26 and OR = 1.6, 95%CI = 0.51–5.3 respectively), though, this was not statistically significant.

**Table 6:** Assessment of maternal physiologic conditions as risk factors for UTI

	Anaemia status		Glocosuria	
	Anaemic	Not anaemic	Present	Absent
**Positive** **n (%)**	12 (32.4)	77 (30.8)	5 (41.7)	84 (30.6)
**Negative** **n (%)**	25 (67.6)	176 (69.2)	7 (58.3)	191 (69.4)
**OR** **(95% CI)**	1.08 (0.51–2.26)		1.6 (0.51–5.3)	
**χ2** **(*p*-value)**	0.04 (0.84)		0.66 (0.41)	

### Performance characteristics of nitrite and leucocyte esterase test

The sensitivity and specificity of both nitrite and leucocyte esterase in the urine dipstick analyses are shown on Table 7. The specificity of the nitrite test was 100% while the sensitivity was very low (4.5%). Leucocytes esterase was more sensitive than it was specific. Using the two tests simultaneously, low sensitivity (50.6%) and specificity (44.9%) values were obtained.

**Table 7:** Validity and reliability of nitrite and leucocytes in screening for UTI

Dipstick test	Individual sens %	Individual spec %	PPV	Net (combined) sens %	Net (combined) spec %
**Leucocyte** **Esterase**	48.3	44.9	40.9	50.6	44.9
**Nitrite**	4.5	**100**	**1**		

*Sens* Sensitivity, *spec* Specificity, *PPV* Positive predictive value

### Antibiotic susceptibility of isolates

The majority of the isolates (75.6%) were most sensitive to ciprofloxacin followed by 70.5%) gentamycin. *E. coli*, the predominant isolate was most sensitive (78.9%) to ciprofloxacin but all (100%) were resistant to ceftriaxone and cefatoxime. Amongst all the gram negative bacteria isolated, *Pseudomonas aeruginosa* had lowest overall sensitivity to all the antibiotics tested even though it was 100% sensitive to gentamycin. All *Staphylococcus aureus* isolates (100%) were sensitive to kanamycin and gentamycin but resistant to ampicillin, cefatoxime and ceftriaxone (Table 8).

**Table 8:** Antibiotic Susceptibility patterns of isolates

	S n (%)	I n (%)	R n (%)	S n (%)	I n (%)	R n (%)	S n (%)	I n (%)	R n (%)	S n (%)	I n (%)	R n (%)	S n (%)	I n (%)	R n (%)
	**K**			**AM**			**CTX**			**CRO**			**C**		
***E. coli***	10 (26.3)	3 (7.9)	25 (65.8)	13 (26.3)	15 (42.1)	10 (31.6)	0 (0)	0 (0)	38 (100)	0 (0)	0 (0)	38 (100)	5 (13.2)	0 (0)	33 (86.8)
***Enterobacter***	3 (100)	0 (0)	0 (0)	0 (0)	0 (0)	3 (100)	0 (0)	0 (0)	3 (100)	0 (0)	0 (0)	3 (100)	0 (0)	0 (0)	3 (100)
***Klebsiella pneumoniae***	0 (0)	10 (58.8)	7 (41.2)	12 (70.6)	5 (29.4)	0 (0)	17 (100)	0 (0)	0 (0)	17 (100)	0 (0)	0 (0)	0 (0)	17 (100)	0 (0)
***Proteus vulgaris***	2 (28.6)	0 (0)	5 (71.4)	2 (28.6)	5 (71.4)	0 (0)	0 (0)	0 (0)	7 (100)	0 (0)	5 (71.4)	2 (28.6)	3 (42.9)	1 (14.2)	3 (42.9)
***Pseudomonas aeruginosa***	0 (0)	0 (0)	5 (100)	0 (0)	0 (0)	5 (100)	5 (100)	0 (0)	0 (0)	0 (0)	0 (0)	5 (100)	0 (0)	3 (60)	2 (40)
***Staphylococcus aureus***	8 (100)	0 (0)	0 (0)	0 (0)	0 (0)	8 (100)	0 (0)	0 (0)	8 (100)	0 (0)	0 (0)	8 (100)	2 (20)	0 (0)	6 (80)
**Total**	**23** **(29.5)**	**15** **(16.7)**	**42** **(53.8)**	**27** **(34.6)**	**25** **(32.1)**	**26** **(33.3)**	**12** **(28.2)**	**0** **(0)**	**56** **(71.8)**	**17** **(21.8)**	**5** **(6.4)**	**56** **(71.8)**	**10** **(12.8)**	**21** **(26.9)**	**47** **(60.3)**
	**CIP**			**CN**			**TE**			**SXT**					
***E. coli***	30 (78.9)	5 (13.2)	3 (7.9)	15 (39.5)	0 (0)	23 (60.5)	5 (13.6)	5 (13.6)	28 (73.7)	3 (7.9)	0 (0)	35 (92.1)			
***Enterobacter***	0 (0)	3 (100)	0 (0)	3 (100)	0 (0)	0 (0)	0 (0)	0 (0)	3 (100)	3 (100)	0 (0)	0 (0)			
***Klebsiella pneumoniae***	17 (100)	0 (0)	0 (0)	17 (100)	0 (0)	0 (0)	0 (0)	7 (41.2)	10 (58.8)	0 (0)	0 (0)	17 (100)			
***Proteus vulgaris***	5 (71.4)	2 (28.6)	0 (0)	7 (100)	0 (0)	0 (0)	0 (0)	0 (0)	7 (100)	2 (28.6)	5 (21.4)	0 (0)			
***Pseudomonas aeruginosa***	2 (100)	0 (0)	0 (0)	5 (100)	0 (0)	0 (0)	5 (100)	0 (0)	0 (0)	0 (0)	5 (100)	0 (0)			
***Staphylococcus aureus***	2 (22)	3 (37.5)	3 (37.5)	8 (100)	0 (0)	0 (0)	5 (62.5)	0 (0)	3 (37.5)	3 (37.5)	0 (0)	5 (62.5)			
**Total**	**59** **(75.6)**	**13** **(18.7)**	**6** **(7.7)**	**55** **(70.5)**	**0** **(0)**	**23** **(29.5)**	**15** **(19.2)**	**12** **(15.4)**	**51** **(65.4)**	**11** **(14.1)**	**10** **(12.8)**	**57** **(73.1)**			

*CN* Gentamycin (20ug), *CIP* Ciprofloxacin (5ug), *SXT* Trimethoprim (1.25ug) and sulphamethoxazole (23.75ug), ***K*** Kanamycin (30ug), *C* chloramphenicol (5ug), *TE* Tetracycline (30ug), *CRO* Ceftriaxone (30ug), *CTX* Cefatoxime (30ug) and *AM* Ampicillin (10 mg). *S* Sensitive, *I* Intermediate, *R* Resistant

## Discussion

Amongst the 287 women surveyed, 89 (31%) had significant bacteriuria. This was higher than the prevalence of 23.5% reported in a similar study in the Buea Health District [10] and 26.1% in Dhaka [16] but lower than 47.5% [17] and 32.7% [18] reported in Nigeria and 35% in India [19]. These differences in prevalence can be attributed to the rate of adherence to ANC visits during pregnancy in the different countries since there is higher probability of being diagnosed and treated for any infection if pregnant women attended ANC as required. It was reported in one of the health areas of the Buea Health District that 84.4% of pregnant women attended at least 4 ANC in the course of pregnancy but only 27.2% booked for ANC in their first trimester [20] which is the trimester with the higher risk of acquiring UTI. The WHO stipulates an 8% reduction in perinatal morbidity and mortality if pregnant women attended up to 8 ANC visits preferably in the 6th, 12th, 20th, 26th, 34th, 36th, 38th and 40th week of pregnancy [21].

Nine bacterial species were responsible for UTIs in our study. *Escherichia coli* was the leading (36.1%) while *Klebsiella oxytoca* was the least (2.4%) cause of UTIs in the participants. Previous studies elsewhere [10, 22–24] have recorded *E. coli* as the leading cause of UTI. Mokube *et al.* [10] also reported *E. coli* as the leading cause of UTI but never detected *Proteus* species in the same study area. Akoachere *et al.* [2] reported *E. coli* as the leading cause of bacteriuria in Buea with no case caused by *Klebsiella pneumoniae.* Changes in aetiologic profile of UTI with time may account for these differences.

The prevalence of ASB (28.7%.) reported in our study greatly exceeds the global range of asymptomatic bacteriuria in pregnancy of 2.5 to 10% [25]. This was higher than 7.8% recorded in Cameroon [10], 23.3% in India [22], 10.7% in Nigeria [23], and 10.4% in Ethiopia [24]. This discrepancy could be related to the fact that most symptoms of UTIs mimic pregnancy discomfort thus neither the patient nor care provider notices the danger signs until the disease develops. For instance, a case review [25], reported preterm rupture of the amniotic sac caused by *Klebsiella pneumoniae* asymptomatic UTI at 32 weeks. This resulted to preterm delivery of a foetus with intrauterine growth retardation. This situation would have been remedied if the pregnant woman was screened for bacteriuria earlier in the pregnancy.

Similar to the findings of Mokube *et al* [10] there was no significant association between maternal obstetric characteristics as risk factors for UTIs in the current study. Contrary to reports elsewhere [16–18, 26, 27], multigravidity, multiparity, gestational age, sexual activity and age amongst other obstetric characteristics have been identified as statistically significant risk factors for UTIs. In our study, the lack of association between maternal obstetric characteristics and prevalence of UTIs was probably due to the differences in characteristics of the study population. In one of the studies [27] where statistically significant associations of UTI were recorded with factors such as advanced age and multiparity, the majority of participants were in the age range 30–40 years and are more likely to have had more than one child unlike in our study were the majority of women were within the age group 21–25 years; within this age group, women are more often, pregnant for the first time, and thus less likely to have been exposed to obstetric situations and conditions than would make them pruned to UTIs.

Early start of ANC (i.e. within the first trimester) increase the chance of being diagnosed of infections early enough and thus avert the probability of carrying infection(s) throughout the course of pregnancy. However, it was observed that, the majority of our study participants started ANC in their first trimester but recorded a greater proportion (41.6%) of UTIs even after the first trimester, although there was no statistical significant association between starting ANC early or late and having UTIs in our study participants. Notwithstanding, starting ANC during the first trimester, as seen with our study participants, is a good practice because all women diagnosed would start treatment early enough and avert any future complications due to UTIs. This also partially explained why there was no significant association between ANC attendance and the occurrence of UTIs recorded in the current study. This good practice of adhering to ANC protocols had been reported in a previous study in the Muea Health Area of the BHD [20] where over 99% of pregnant women attended at least one ANC visit and 84.8% attended at least four visits.

With respect to personal hygiene, there was no significant association between the type of toilet used or type of pant wore with the occurrence of UTIs in pregnancy. This was contrary to the findings of Ahmad and colleagues [27] who reported a statistically significant risk of the occurrence of UTIs when pants made of silk were put on, compared with those wore pants made of cotton. The difference in the findings of Ahmad and colleagues from the current study might be due to the fact that some women in our study reported not wearing pants of any type at all, due to a discomforting feeling, especially those approaching end of term. This could be the cause of variations in reports even from other studies carried out in our setting.

Regarding the influence of other health conditions of the women, no significant association was recorded between glucosuria, anaemia or previous UTIs and UTIs in pregnancy. However, our analyses showed that the presence of glucose in urine and being anaemic increased the risk of acquiring UTIs in pregnancy (OR = 1.6, 95%CI = 0.51–5.3, OR = 1.08, CI = 0.51–2.26 respectively) (*p* = 0.41 and *p* = 0.84 respectively). Haider *et al*. [26] also recorded being anaemic as an influencer of UTI. Other researchers like Ahmed *et al*. [28] reported hyperglycemia as an important risk factor of UTIs in Saudi Arabia unfortunately, this study only measured glucosuria which is not suitable enough for the diagnosis of gestational diabetes. Adherence to ANC protocols recorded in our study could be the reason for the lack of a statistically significant association since pregnant women who attend ANC regularly are screened for glucosuria and are placed on daily consumption of iron supplement. The situation might have been different if this was a community based study that would have included women who do not attend ANC at all.

The combii 11 dipstick test revealed a very low sensitivity (4.5%) and an absolute (100%) specificity for nitrite with a positive predictive value of 1. In other words, if someone shown positive for UTI by a positive nitrite dipstick, then that person was truly having a UTI. However, values also indicate that, the test would totally fail to detect UTIs in those who are negative for nitrite. On the other hand, leucocyte esterase had a fairly low sensitivity of 48.3% and specificity 44.9%. The net sensitivity (50.6%) and specificity (44.9%) of both tests used simultaneously were low. These values indicated that, it was preferable to confirm infection with the nitrite test alone than with both nitrite and leucocyte esterase together. However, employing both tests together with signs/symptoms such as haematuria and moderately severe dysuria could be a good and cost effective test for the screening of UTIs in pregnancy, since it increases the sensitivity and specificity by 80 and 54% respectively as previously reported [29]. Mokube *et al.* [10] reported a much higher sensitivity of 8% for nitrite and lower sensitivity 20.8% for leucocyte esterase. They reported a lower specificity for nitrite (98.7%) compared to our absolute value (100%) while the leucocyte esterase specificity value (80.8%) was much higher than the 44.9% recorded in the current study. These variations, especially with the nitrite could account for the differences in the number and types of bacterial isolates recorded in different studies on pregnant women. Given that Gram positive organisms do not reduce nitrate to nitrite in urine, the lower the sensitivity of nitrite as a screening test was understandable. For leucocytes, the source of leucocytes in urine could not be ascertained considering solely the data collected during the current study, since other sexually transmissible infections, such as *Chlamydia trachomatis*, which constitute a health challenge in Cameroon [30] also shed leucocytes into urine.

Isolates from our study were most sensitive to ciprofloxacin and gentamycin (75.6%) contrary to those found in the study by Mokube and colleagues [10] who recorded the highest sensitivity to the cephalosporins; cefixime and cefoxitin in the same study area. Corroborating our findings, Akoachere *et al.* [2] reported gentamycin as the most active drug for empirical treatment of UTIs in Buea. A similar observation was reported in Bamenda Cameroon [31]. Ampicillin had been a drug of choice for the treatment of UTIs until increasing resistance of *E. coli*, the leading cause of UTIs, to this drug was reported [6]. This could be the reason for the fairly low susceptibility of this drug (34.6%) to the uropathogens tested in the current study. Contrary to this finding, Akoachere *et al.* [2] and Mokube *et al.* [10] recorded a considerable susceptibility of uropathogens to ampicillin in Buea. However, their studies, involved participants other than pregnant women. Given that only a limited number of antibiotics are safe for pregnant women, overuse of a particular antibiotic because of its safety is imminent, which might lead to resistance thus the differences recorded in susceptibility pattern reported with isolates from the same study area.

## Conclusions

From our findings, the prevalence of UTIs in pregnant women in the Buea Health District (BHD) was quite high. However, we identified no significant risk factors associated with these infections thus making it a necessary recommendation for pregnant women to be screened for UTIs at least once during pregnancy and positive cases treated to prevent the effects of this infection on pregnancy outcome. A longitudinal study on this subject, with a larger sample size would be ideal in order to follow up positive women to term, to identify the gravity of this infection on pregnancy outcome. To avoid an undesirable pregnancy outcome, the Combii 11 should be used for monthly routine ANC checkups rather than the Combii 2 such that any nitrite positive case can be treated directly since the nitrite was seen to be 100% specific. On the other hand, pregnant women with symptoms or with considerable concentration of leucocyte esterase in urine should be subjected to culture for a very specific treatment to be administered. The recorded susceptibility of uropathogens to ciprofloxacin and gentamycin suggests these antibiotics for empirical treatment of UTIs in the BHD, with medical advice on its safety from an obstetrician.

## Data Availability

All data generated or analyzed during this study are included in the published article.

## Abbreviations

UTI: Urinary Tract Infection
ASB: Asymptomatic Bacteriuria
BHD: Buea Health District
ANC: Antenatal Consultation
